# Incorporating Treatment Time into Butterfly Optimization to Reduce Total Treatment Time for Vaginal Cylinder Brachytherapy

**DOI:** 10.7759/cureus.23893

**Published:** 2022-04-06

**Authors:** Xingen Wu, Ivan A Brezovich, Sui Shen, Elizabeth Covington, Dennis Stanley, Richard Popple

**Affiliations:** 1 Radiation Oncology, University of Alabama at Birmingham School of Medicine, Birmingham, USA; 2 Radiation Oncology, University of Michigan, Ann Arbor, USA

**Keywords:** radiotherapy (rt), optimization, meta-heuristic algorithm, butterfly algorithm, hdr (high dose rate) brachytherapy

## Abstract

Purpose

For patient comfort and safety, irradiation times should be kept at a minimum while maintaining high treatment quality. In this study of high dose rate (HDR) therapy with a vaginal cylinder, we used the butterfly optimization algorithm (BOA) to simultaneously optimize individual dwell times for precise dose conformity and for the reduction of total dwell time.

Material and methods

BOA is a population-based, meta-heuristic algorithm that averts local minima by conducting intensive local and global searching based on switching probability. We constructed an objective function (a stimulus intensity function) that consisted of two components. The first one was the root-mean-squared dose error (RMSE) defined as the square root of the sum of squared differences between the prescribed and delivered dose at the constraint points. The second component was weighted total treatment time. Eight previously treated cases were retrospectively reviewed by re-optimizing the clinical treatment plans with BOA.

Results

Compared to the eight original plans generated with the commercial adaptive volume optimization algorithm (AVOA), the BOA-optimized plans reduced treatment times by 5.4% to 8.9%, corresponding to a time-saving of 13.1 to 47.7 seconds with the activities on the treatment day and saving from 29.3 to 64.6 seconds if treated with an activity of 5 CI. Dose deviations from the prescription were smaller than in the original plans.

Conclusion

Dose optimizations based on the BOA algorithm yield closer dose conformity in vaginal HDR treatment than AVOA. Incorporating total treatment time into the optimization algorithm reduces the delivery time while having only a small effect on dose conformity.

## Introduction

High dose rate (HDR) brachytherapy with vaginal cylinders is a standard modality for treating low-risk endometrial cancer as monotherapy and as a boost to external beam radiation for high-risk cancer. According to the American Brachytherapy Society Consensus Guidelines, the vagina at risk needs proper coverage for 3 to 5 cm along the cylinder, with the dose prescribed at the applicator surface or 0.5 cm depth from the surface [1]. Treatment plans find source dwell times that yield dose distributions that most closely match the prescription [2-9].

Many optimization algorithms have been suggested for determining the required dwell times. Li et al. studied the effects of prescription depth, cylinder size, treatment length, tip space, and curved end [6]. Supe et al. compared two different dose optimization methods: non-apex (optimizing points only on the periphery of the cylinder) and apex (optimizing points on the periphery and along the rounded apex) [2]. They found the dose distribution to be more uniform when the apex points were included in the optimization [3]. Alterovitz et al. used linear programming for treatment planning and compared it to simulated annealing [4]. Carrara et al. compared the dosimetric performance of different forward and inverse treatment planning methods of multi-channel vaginal cylinders, including dose-point optimization followed by graphical optimization, inverse planning simulated annealing with two different class solutions as starting conditions, and hybrid inverse planning optimization [5]. A literature review of optimization methods can be found in Reddy’s publication [10]. 

While the above-mentioned treatment planning methods optimize dose conformity, they do not consider delivery efficiency. In this paper, we present a novel strategy for treatment planning that includes treatment time as an optimization goal. Our optimization uses BOA, a published metaheuristic for global optimization [11,12]. In addition, BOA was compared to other metaheuristic optimization algorithms, including Cuckoo search, artificial bee colony, differential evolution, and genetic algorithms, and was found superior in most benchmark cases [11]. We demonstrate the benefit of our strategy by using eight previously treated cases of vaginal HDR irradiation. We compare the treatment times and dose conformity of our method to the historical cases that used the adaptive volume optimization algorithm (AVOA).

## Materials and methods

BOA simulates the foraging behavior and information-sharing strategy of butterflies. It conducts a search by simulating the collective movement of butterflies toward food sources for mating positions by receiving, sensing, and analyzing the smell [11,12]. The "fragrance (f)" of a butterfly is fitness or an objective function of sensory modality $c$, stimulus intensity $I$, and power exponent $\alpha$. The three components decide the change of fragrance in a linear or non-linear response. As a population-based global optimization algorithm, BOA maintains a fixed population during a search, and its individual agents interact with each other and trace out multiple paths to select the best solution. For vaginal cylinder treatment planning, we define the $i^{th}$ butterfly as a possible dwell solution (a vector): 

$B_{i}=(T_{i,1}, T_{i,2}, ..., T_{i,M})$* *(1)

where $M$ is the number of dwell positions and $i = 1, 2, ..., P$, with $P$ defined as the population size. The stimulus intensity $I_{i}$ of the $i^{th}$ butterfly is the sum of the RMSE and a weighted total dwelling time,

$I_{i}=\sqrt{\sum_{j=1}^{N} \frac{w_{j}(D_{i,j}-D)^{2}}{N}} + \beta\sum_{k=1}^{M} T_{k}$ (2)

where *N* is the number of the constrain points. $w_{j}$ is the weighting factor for constraint point $j$. $D$ is the prescription dose and $T_{k}$ is the dwell time at the $k^{th}$ position. $D_{i,j}$ is the dose at the $j^{th}$ constraint point calculated from the current dwelling positions, and $\beta$ is the weighting factor for the total dwell time. In the search for the global minimum (best solution), $\beta$ is adjusted for a good compromise between dose conformity and short treatment time. The fragrance $f_{i}$ of $i^{th}$ butterfly is defined as:

$f_{i}=cI_{i}^{\alpha }$* *(3)

where $c$ is the sensory modality and $\alpha$ is the power exponent. The values of these two coefficients lie between 0 and 1. The BOA algorithm is composed of three phases: initialization, iteration, and final phase. A detailed description can be found in reference [11].

## Results

All dose calculations were based on the American Association of Physicists in Medicine Task Group 43 report [13]. We planned the clinical cases using BrachyVision (version 15.5.11, Varian Medical System, Palo Alto, CA), a system based on the adaptive volume optimization algorithm (AVOA). Radiation was delivered with the afterloading system VariSource IX (Varian Medical System, Palo Alto, CA). Our BOA-based computations are ranging on an HP desktop (Intel core i-i5-8500 CPU @ 3.0 GHz). Following optimization, we used BrachyVision to calculate doses based on our optimized dwell times. So, computed doses were used to find the RMSEs at the constraint points.

The clinical cases consisted of seven endometrial adenocarcinomas (cases 1-7) treated with 21 Gy in three fractions, prescribed at a 5 mm depth from the applicator surface. The eighth case (case 8) involved a vaginal cuff brachytherapy boost to 12 Gy in two fractions, prescribed at the cylinder surface. For each case, two digital imaging and communications in medicine (DICOM) files (the plan file and structure file) were exported from the Eclipse planning system and were read into the home-developed BOA software written in MATLAB and C++ language. The constrain points on the reference lines (5 mm from the applicator surface for the first seven cases or on the surface lines in the last case) were used in the optimization as the treatment target with the prescription dose. 

For all eight cases, the population was set to 80 and the maximum iteration to 5,000 times. The program ran on a Windows desktop. The sensory modality $c$ was set to 0.2 and the initial value of power exponent $\alpha$ was set to 0.03. For the constrain points at the top of the applicator, the weighting factor $w_{j}$ is set to 1.2 due to the anisotropic function at the applicator's top direction. The factor $\beta$ was set to 0.6 to balance the two terms in equation (2). The optimization took less than one minute.

Table 1 shows a comparison of the eight clinical cases originally optimized with AVOA and retrospectively re-optimized with BOA. The first dwell position is at the tip of the vaginal cylinder. The following positions are spaced in 2.5 mm increments. BOA shortens the total dwell time by 13.1 seconds to 47.7 seconds, corresponding to 5.4% to 8.9% of the total treatment time. If the patients were treated at an activity of 5 CI, the treatment time could be saved from 29.3 seconds to 64.6 seconds. The last column shows the RMSE is consistently lower for BOA. 

**Table 1:** Comparison of eight treatment plans between AVOA and BOA. AVOA: adaptive volume optimization algorithm; BOA: butterfly optimization algorithm; RMSE: root-mean-squared dose error.

Case number	Optimization methods	Relative dose on the prescription lines (%)			Total dwelling time (s)	Difference		Difference if projected to 5 CI	RMSE
		Max	Min	Mean		S	%		
1	AVOA	112.5	92.4	100.5	429.2	23.8	5.5	29.3	24.2
	BOA	110.3	92.5	100.1	405.4				16.4
2	AVOA	112.7	93.2	100.9	571.5	47.7	8.3	64.6	30.2
	BOA	111.5	94.1	100.5	523.8				15.4
3	AVOA	123.6	95.4	102.4	501.1	28.1	5.6	40.6	37.9
	BOA	119.4	94.7	101.2	473.0				20.4
4	AVOA	115.3	92.7	100.6	521.6	42.1	8.1	36.8	28.6
	BOA	116.4	93.3	101.1	479.5				20.3
5	AVOA	115.7	93.9	101.2	384.5	31.3	8.1	46.8	30.3
	BOA	112.5	93.2	100.8	353.2				23.1
6	AVOA	121.1	94.6	101.4	416.4	36.9	8.9	51.7	25.6
	BOA	119.8	93.2	101.1	379.5				17.4
7	AVOA	116.3	92.5	101.1	423.5	26.8	6.3	36.8	32.5
	BOA	117.4	93.2	101.3	396.7				17.9
8	AVOA	112.7	92.1	100.2	242.8	13.1	5.4	31.8	25.3
	BOA	110.4	92.3	100.3	229.7				20.1

**Figure 1:**
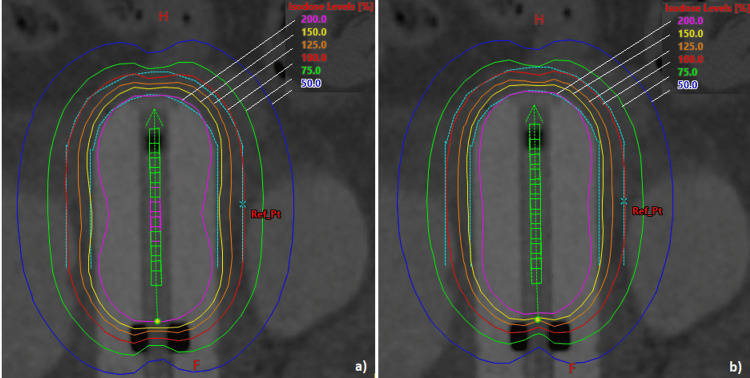
Comparison of isodose lines in the central plane, case 1: (a) AVOA, (b) BOA. AVOA: adaptive volume optimization algorithm; BOA: butterfly optimization algorithm.

Figure 1 compares isodose lines for case 1 computed from AVOA and BOA produced dwell times. Isodose lines from 50% to 200% are displayed in planes containing the axis of the vaginal cylinder. 

**Figure 2:**
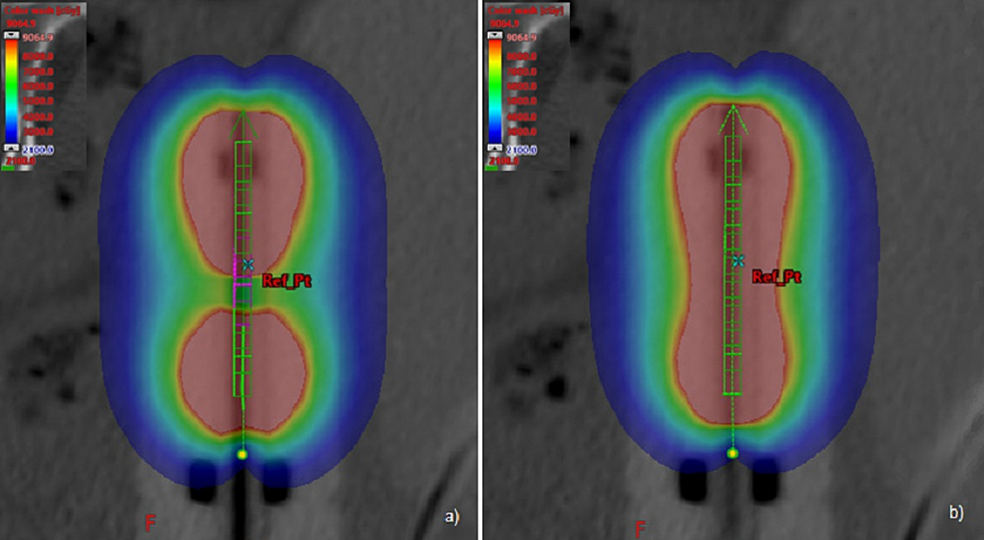
Comparison of absolute doses in the central plane, case 2: (a) AVOA and (b) BOA. AVOA: adaptive volume optimization algorithm; BOA: butterfly optimization algorithm.

Figure 2 shows a similar comparison for case 2, depicting absolute doses in color-wash. 

## Discussion

The proper choice of $\beta$ is important because it balances the two competing objectives of accurate dose conformity and short treatment time. Under an extreme condition of $\beta$=0, the optimization will search for the best solution without considering any time-saving. The value of $\beta = 0.6$ used in this study was found by trial and error. Power exponent $\alpha$ is a dynamic parameter and it is linearly increased from its initial value of 0.03 to 0.3 with iterations in order to increase the degree of fragrance absorption $f$.

The computation time of the BOA generated on an HP desktop computer without a graphic processing unit (GPU) was slightly longer (about one minute) than that of the AVOA, which was running on a multi-core (16) GPU workstation server (less than five seconds). Unfortunately, we could not replicate the AVOA (like a black box) and run it on our desktop computer to have a fair comparison with BOA. 

In all eight cases, we didn't have the dose constraints for organs-at-risk in the optimization because the dose was prescribed either on the cylinder surface or at the 5 mm depth away from the cylinder surface. The dose to the regional organs, like the rectum, small bowel, and bladder, is relatively small, and complications are rare in our institution. We listed the relative maximum, minimum, and mean doses on the prescribed lines (see Table 1, column 3 to column 5). They were normalized to the prescription dose. There is no meaningful difference between AVOA and BOA, showing the BOA plans can deliver the same expected dose distribution while reducing the total delivery time at the same time.

The current commercial brachytherapy treatment planning systems are focused on conformity for desired dose distribution. Treatment time is not considered as one of the optimization goals [14-16]. In this study, the total treatment time was brought into the optimization with other optimization goals for the first time by using a global optimization method, BOA. As a metaheuristic optimization method, BOA demonstrated that it is superior in most benchmark optimization cases [11] and can effectively avoid local extrema in searching for the best dwelling time for each dwelling position. 

All the radiation treatment procedures are huge challenges for our patients, especially for gynecological brachytherapy treatment. Any measure to shorten this process can reduce the patient's pain. Incorporating the total treatment time into the optimization was first proposed in this study, and the result is encouraging, although the total treatment time was saved roughly by 5% to 8%. The main reason is that the treatment itself-most vaginal cylinder brachytherapy only has one channel. Applying this method to multiple channel cases like tandem and ovoid, interstitial multiple needles will be our next step. We also plan to apply this method to external treatment planning to reduce the total treatment time by considering the combination of couch angles, gantry rotation, and dose rate in the cost function of optimization algorithms. While maintaining the same or similar treatment goals of dose coverage for the treatment target and dose constraints to the surrounding critical organs, reducing the total treatment time is helpful to minimize the patient's motion during the treatment and make the patient more comfortable during the treatment.

## Conclusions

Dose optimizations based on the BOA algorithm yield closer dose conformity in vaginal HDR treatment than AVOL optimization. Incorporating total treatment time into the optimization algorithm reduces the delivery time while having only a small effect on dose conformity. The small increase in planning time is offset by shorter treatment times. Considering that patient motion is detrimental to accurate dose delivery, shortening treatment times can improve quality and patient throughput. Such an improvement could be especially beneficial in intensity-modulated radiation therapy with high modulation. Long treatment times can lead to patient motion and geographic misses, while large numbers of monitor units expose patients to increased doses from radiation leakage and collimator scatter.
